# The emerging role of dendritic cells in the host immune response against *Helicobacter pylori*

**DOI:** 10.3389/fmicb.2014.00560

**Published:** 2014-10-24

**Authors:** Steve Oghumu, Abhay Satoskar

**Affiliations:** ^1^Department of Pathology, Ohio State University Medical CenterColumbus, OH, USA; ^2^Department of Oral Biology, Ohio State University College of DentistryColumbus, OH, USA

**Keywords:** *Helicobacter*, cytotoxin, microRNA, adenocarcinoma, dendritic, cytokine

Host cell interactions with bacterial pathogens trigger a wide variety of complex cellular signaling pathways that ultimately determine disease outcome. These inflammatory signaling cascades can often be traced back to specific virulence factors within the pathogen, which interact with unique host cells that come in contact with the invading organism. *Ex vivo* model systems which recapitulate the key events of human infection have helped to clarify these signaling activities with the critical molecules involved in the process. This was exemplified in the study conducted by Hocès de la Guardia et al. (2013) on *Helicobacter pylori*, which causes chronic active gastritis and peptic ulcer disease in humans. *H. pylori* infection has also been considered to be a risk factor for the development of gastric adenocarcinoma and mucosa-associated lymphoid tissue (MALT) lymphoma in some patients (Parsonnet et al., 1991, 1994). Type I strains contain the virulence factor cytotoxin-associated gene A (CagA), which has been shown to mediate the pathology associated with intestinal disease caused by *H. pylori* (Censini et al., 1996).

Studies evaluating pro-inflammatory immune responses against *H. pylori* infection have mainly been based on *in vitro* models using the AGS gastric epithelial cell line. However, it is becoming increasingly clear that interactions between pathogen and gut associated immune cells, particularly dendritic cells (DCs), play a major role in directing the nature of the adaptive immune response against *H. pylori* (Shiu and Blanchard, 2013). It is therefore critical to define mechanisms by which *H. pylori* modulates DC function. Interactions between *H. pylori* and DCs occur either in the gut lumen where mucosal DCs insert dendrites through the tight junctions of the gut epithelial monolayer (Rescigno et al., 2001), or within Peyer's patches in the small intestine where resident DCs phagocytose bacteria (Nagai et al., 2007). Mediated primarily by Toll-like receptors (TLRs) expressed on their cell membrane, DCs recognize pathogen associated molecular patterns (PAMPs) present on *H. pylori*, an interaction which triggers host cell signaling cascades that are vital for the initiation of the host immune response (Rad et al., 2009; Kabisch et al., 2014; Smith, 2014). Consistent with previous findings, the study by Hocès de la Guardia et al. (2013) observed enhanced production of TNFα, IL-6 and IL-10 by *H. pylori* infected DCs (Rad et al., 2009). In contrast with *Escherichia coli*-derived LPS (which signals primarily through TLR4), production of these cytokines was delayed in *H. pylori* infected DCs, suggesting differential mechanisms of activation, possibly through TLR8 and TLR9 signaling pathways. Moreover, production of IL-10 was significantly enhanced in DCs co-cultured with *H. pylori*, which fosters an anti-inflammatory microenvironment mediated by signaling through TLR2 and DC-SIGN (Rad et al., 2009; Fehlings et al., 2012). These important findings support the notion that pathogen-derived factors are important modulators of the host immune response which ultimately affects the outcome of disease.

An important finding by the authors of this study was the preferential induction of the cytokine IL-23 by DCs cultured with *cag* Pathogenicity Island (*cag*PAI)-containing *H. pylori* strains. While some papers report similar findings (Khamri et al., 2010; Tanaka et al., 2010), others indicate that cytokine production by *H. pylori*-infected DCs is cagPAI independent (Kao et al., 2010; Horvath et al., 2012). Additional studies will be required to clarify the effect of *cag*PAI in skewing the host immune response against *H. pylori*. It is possible that the type of DCs, bacterial strains and experimental conditions utilized affect the results obtained in these various studies. Nevertheless, the ability of *H. pylori* to induce IL-23 production in cultured DCs has been validated by this study. The production of IL-23 by *H. pylori* infected DCs has potential implications in the induction and maintenance of Th-17 responses, which could affect the development of gastritis during *H. pylori* infection.

The studies conducted by Hocès de la Guardia et al. (2013) further showed a temporal regulation of miRNAs (miR-146a and miR-155) by *H. pylori* which, although not dependent on the *cag*PAI, significantly affected the expression of pro-inflammatory cytokines (TNFα and IL-10). A role for these miRNAs (particularly miR-155) in fine tuning of TNF- and NFκ B-mediated inflammatory responses was well established by loss-of-function assays using antisense miRNAs which inhibit the activity of miR-146a and miR-155. These miRNAs have previously been linked to chronic inflammatory diseases (Sonkoly and Pivarcsi, 2009) and lymphoma development (Costinean et al., 2006). It is therefore of great interest to investigate how the deregulation of miR-146, miR-155 and other miRNAs contributes to the pathologies and complications associated with *H. pylori* infection.

The article by Hocès de la Guardia et al. (2013) has significantly impacted the field by clearly defining *H. pylori* mediated inflammatory response modulation using an *ex vivo* model system of primary human DCs. Using this *ex vivo* co-culture system, the authors were able to establish a role for *H. pylori cag*PAI in directing the host adaptive immune response toward a Th-17 profile by inducing IL-23 production in DCs. Therapeutic approaches that target key host response mediators modulated by *H. pylori* could subsequently be exploited in the management of *H. pylori* infection and its associated pathologies.

## Conflict of interest statement

The authors declare that the research was conducted in the absence of any commercial or financial relationships that could be construed as a potential conflict of interest.
